# An evaluation *in vitro* of the efficacy of nutlin-3 and topotecan in combination with ^177^Lu-DOTATATE for the treatment of neuroblastoma

**DOI:** 10.18632/oncotarget.25607

**Published:** 2018-06-26

**Authors:** Mathias Tesson, Richa Vasan, Andreas Hock, Colin Nixon, Colin Rae, Mark Gaze, Robert Mairs

**Affiliations:** ^1^ Radiation Oncology, Institute of Cancer Sciences, Wolfson Wohl Translational Cancer Research Centre, University of Glasgow, Bearsden, Glasgow, UK; ^2^ Cancer Research UK Beatson Institute, Bearsden, Glasgow, UK; ^3^ Department of Oncology, University College London Hospitals NHS Foundation Trust, London, UK

**Keywords:** DOTATATE, neuroblastoma, radiosensitisation, topotecan, nutlin-3

## Abstract

Targeted radiotherapy of metastatic neuroblastoma using the somatostatin receptor (SSTR)-targeted octreotide analogue DOTATATE radiolabelled with lutetium-177 (^177^Lu-DOTATATE) is a promising strategy. This study evaluates whether its effectiveness may be enhanced by combination with radiosensitising drugs. The growth rate of multicellular tumour spheroids, derived from the neuroblastoma cell lines SK-N-BE(2c), CHLA-15 and CHLA-20, was evaluated following treatment with ^177^Lu-DOTATATE, nutlin-3 and topotecan alone or in combination. Immunoblotting, immunostaining and flow cytometric analyses were used to determine activation of p53 signalling and cell death. Exposure to ^177^Lu-DOTATATE resulted in a significant growth delay in CHLA-15 and CHLA-20 spheroids, but not in SK-N-BE(2c) spheroids. Nutlin-3 enhanced the spheroid growth delay induced by topotecan in CHLA-15 and CHLA-20 spheroids, but not in SK-N-BE(2c) spheroids. Importantly, the combination of nutlin-3 with topotecan enhanced the spheroid growth delay induced by X-irradiation or by exposure to ^177^Lu-DOTATATE. The efficacy of the combination treatments was p53-dependent. These results indicate that targeted radiotherapy of high risk neuroblastoma with ^177^Lu-DOTATATE may be improved by combination with the radiosensitising drugs nutlin-3 and topotecan.

## INTRODUCTION

Neuroblastoma is a malignancy predominantly of infancy and young children. It originates most commonly in the adrenal glands [1] and affects about one hundred individuals per year in the UK (http://www.cancerresearchuk.org). About half of neuroblastomas are highly aggressive, as indicated by dissemination in children over one year of age or by adverse biological features such as amplification of the MYCN oncogene and by unresponsiveness to induction therapy or early relapse if a response is achieved. These high-risk neuroblastomas are responsible for 15% of paediatric cancer fatalities and new treatments are urgently needed [1]. Ninety percent of neuroblastoma tumours express the noradrenaline transporter (NAT). These can be treated with molecular radiotherapy using the iodine-131-radiolabelled noradrenaline analogue meta-iodobenzylguanidine (^131^I-mIBG) [2]. Targeted radiotherapy of neuroblastoma using ^131^I-mIBG has produced encouraging long-term remission and palliation [3]. However, not all neuroblastoma tumours express NAT and resistance to ^131^I-mIBG therapy may occur [3]. This has encouraged the consideration of alternative radiopharmaceuticals for the targeted treatment of neuroblastoma. Somatostatin receptors (SSTRs) are overexpressed in human neuroblastomas [4–6] and the lutetium-177-radiolabelled somatostatin analogue DOTATATE (^177^Lu-DOTATATE) binds with high affinity to SSTRs, particularly subtype 2 (SSTR2). The efficacy and safety of ^177^Lu-DOTATATE therapy in neuroendocrine tumours have been reviewed elsewhere [7]. Its therapeutic application in neuroblastoma is appropriate particularly for patients whose tumours demonstrate good uptake of ^68^Ga-DOTATATE on PET/CT imaging, who do not have a positive ^123^I-mIBG diagnostic scan or whose tumours are unresponsive to, or relapse after, ^131^I-mIBG therapy.

The safe and successful treatment of children with neuroblastoma using ^177^Lu-DOTATATE was recently reported [8, 9]. However, it is expected that maximal therapeutic potency of targeted radiotherapy will be derived from its combination with radiosensitisers [10, 11]. We previously showed that the topoisomerase I inhibitor topotecan enhanced ^131^I-mIBG efficacy in preclinical models [12]. Based on this study, the feasibility and tolerability of ^131^I-mIBG treatment in combination with topotecan were evaluated in children with metastatic neuroblastoma [13] and, subsequently, a prospective phase II study reported an overall response rate of 57% [14]. Finally, the multicentre randomised SIOPEN study EudraCT N° 2015-003130-27 will compare the effectiveness of ^131^I-mIBG in combination with topotecan to that of high dose thiotepa.

An alternative radiosensitising strategy is indicated by consideration of the role played by p53 in the response of neuroblastoma tumours to cytotoxic therapy. In neuroblastoma, the impairment of p53 signalling is seldom caused by inactivating mutations of the p53 gene [15, 16] but is more commonly due to upstream aberrations such as amplification of the p53 negative regulator MDM2 and inactivation of the MDM2 inhibitor p14(ARF) [17]. Genetic studies have indicated that the restoration of p53 activity has promising therapeutic potential [18, 19], notably through induction of apoptosis and senescence in tumour cells [20, 21]. For instance, the cis-imidazoline small molecule nutlin-3 binds to the p53-binding pocket of MDM2, stabilises p53 and reduces tumour cell viability [22]. It has also been shown to enhance the efficacy of targeted radiotherapy [23] and to sensitise tumour cells to radiation treatment via p53-mediated apoptosis [24] or senescence [25]. Interestingly, it has been reported that resistance of tumour cells to the cytotoxicity of p53 restoration could be overcome by radiation treatment [26]. Finally, the activation of the HIPK2 and p53 axis by nutlin-3 specifically in MYCN-amplified tumours sensitises tumours to apoptosis [27], thereby supporting that the strategy consisting of reactivating p53 in combination with radiotherapy is suitable for the treatment of patients with high risk neuroblastoma.

Topotecan has been shown to induce p53 expression [28, 29] and to synergise with MDM2 inhibitors *in vitro* and *in vivo* [30, 31]. It has also been suggested that nutlin-3 may enhance the efficacy of chemotherapy [32, 33], notably through inhibition of multi-drug resistance protein 1 function [34]. Therefore, while others have demonstrated the benefit to be derived by the combination of nutlin-3 and topotecan, we hypothesised that this combination may sensitise neuroblastoma cells to ^177^Lu-DOTATATE targeted radiotherapy in a manner analogous to that of topotecan blended with ^131^I-mIBG treatment [12].

The aims of this study were first to characterise neuroblastoma cell lines with respect to their sensitivity to X-irradiation and ^177^Lu-DOTATATE as well as their ability to activate p53 signalling following treatment with nutlin-3, topotecan or X-irradiation; and second to assess whether the combination treatment consisting of topotecan and nutlin-3 sensitised neuroblastoma cells to X-irradiation and ^177^Lu-DOTATATE treatment and the relationship with activation of p53 signalling.

## RESULTS

### Characterisation of the sensitivity of SK-N-BE(2c), CHLA-15 and CHLA-20 spheroids to ^177^Lu-DOTATATE treatment

**Figure 1:**
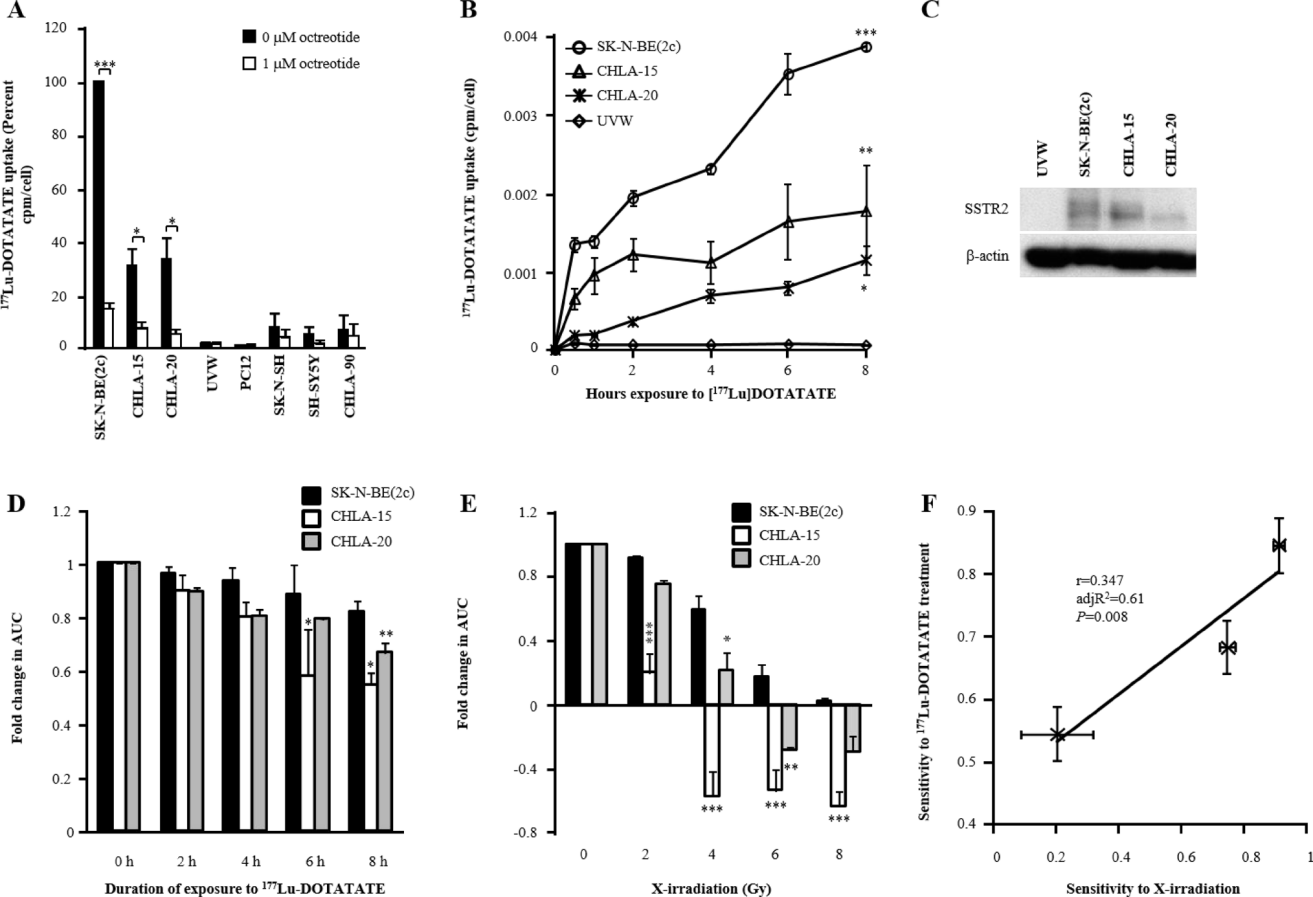
The effect of exposure of SSTR2-expressing cells to ^177^Lu-DOTATATE on its intracellular accumulation and spheroid growth delay (**A**) SSTR-mediated uptake of ^177^Lu-DOTATATE by various cell lines was measured after 4 h incubation with 100 KBq/ml ^177^Lu-DOTATATE in the presence or in the absence of 1 μM octreotide. Data are means ± SEM, *n* = 3. Paired-samples *t*-tests were performed to determine the significance of the reduction in uptake by octreotide. (**B**) The cytosolic accumulation of ^177^Lu-DOTATATE was measured over 8 h following treatment with 100 kBq/ml ^177^Lu-DOTATATE. Data are means ± SEM, *n* = 3. Bonferroni-corrected one-way ANOVA was performed. *P*-values indicate the significance of the difference between the mean uptake values at 8 h of SK-N-BE(2c), CHLA-15 and CHLA-20 cells in comparison with that of UVW cells. (**C**) The expression of SSTR2 was evaluated by immunoblotting in SK-N-BE(2c), CHLA-15 and CHLA-20 cells with UVW cells as negative control and β-actin as a loading control. (**D**) The spheroid growth delay induced by treatment of SK-N-BE(2c), CHLA-15 and CHLA-20 spheroids with 5 MBq/ml ^177^Lu-DOTATATE for 8 h is reported as a decrease in AUC values. The radioactivity concentration 5 MBq/ml was chosen based on preliminary experiments. Data are mean ± SEM, *n* = 3. One-way ANOVA with Bonferroni correction was performed. *P*-values indicate the significance of the difference in AUC values in comparison with that of untreated spheroids for each cell line. (**E**) The radiosensitivity of SK-N-BE(2c), CHLA-15 and CHLA-20 spheroids. Data are mean ± SEM, *n* = 3. One-way ANOVA with Bonferroni correction was performed. At each radiation dose, *P*-values indicate the significance of the difference in AUC values in comparison with that of SK-N-BE(2c) spheroids. (**F**) The AUC values of SK-N-BE(2c), CHLA-15 and CHLA-20 spheroids following treatment with 5 MBq/ml ^177^Lu-DOTATATE for 8 h correlated with AUC values obtained following treatment with 2 Gy X-irradiation. Data are mean ± SEM, *n* = 3. In all panels, one symbol indicates *P* < 0.05, two symbols indicate *P* < 0.01 and three symbols indicate *P* < 0.001.

Uptake assays were performed in monolayers of various cell lines to determine their ability to concentrate ^177^Lu-DOTATATE intracellularly. The competitive inhibitor of binding to SSTR, octreotide, was used to determine whether binding of ^177^Lu-DOTATATE to SSTR was required for intracellular transport. There was significantly greater ^177^Lu-DOTATATE internalisation by SK-N-BE(2c) (*P* < 0.001), CHLA-15 (*P* < 0.05) and CHLA-20 (*P* < 0.05) cells following exposure to ^177^Lu-DOTATATE compared with exposure to ^177^Lu-DOTATATE in the presence of 1 μM octreotide (Figure 1A). In contrast, UVW, PC12, SK-N-SH, SH-SY5Y and CHLA-90 cells did not internalise ^177^Lu-DOTATATE (Figure 1A). Furthermore, there was a statistically significant, time-dependent, accumulation of ^177^Lu-DOTATATE by SK-N-BE(2c) (*P* < 0.001), CHLA-15 (*P* < 0.01) and CHLA-20 cells (*P* < 0.05), but not by UVW cells (Figure 1B). This observation is consistent with the expression of SSTR2 by SK-N-BE(2c), CHLA-15 and CHLA-20 cells, but not by UVW cells (Figure 1C). Immunoblotting analysis of SSTR2 revealed variation in the apparent molecular size of SSTR2, as indicated by a broad band (Figure 1C). This has been hypothesised previously to be due to post-translational modifications [35, 36]. Finally, a growth delay mediated by 8 h exposure to ^177^Lu-DOTATATE was indicated by a 1.2-fold (not significant), 1.9-fold (*P* < 0.05) and 1.5-fold (*P* < 0.01) decrease in AUC values in spheroids derived from SK-N-BE(2c), CHLA-15 and CHLA-20 cells, respectively (Figure 1D, Supplementary Figure 1A), suggesting that SK-N-BE(2c) spheroids were more resistant to ^177^Lu-DOTATATE than CHLA-15 and CHLA-20 spheroids. This may be explained by their relative radiosensitivity. Indeed, SK-N-BE(2c) spheroids were significantly more resistant to 4 or 6 Gy X-irradiation than either CHLA-15 or CHLA-20 spheroids (Figure 1E, Supplementary Figure 1B). Moreover, the fold change in AUC in response to ^177^Lu-DOTATATE treatment correlated with that obtained in response to X-irradiation (*r* = 0.347, adj *R*^2^ = 0.61, *P* = 0.008) (Figure 1F). Together, these results indicated that SK-N-BE(2c), CHLA-15 and CHLA-20 spheroids were suitable *in vitro* experimental models to assess SSTR2-targeted therapy. Furthermore, the sensitivity of spheroids to ^177^Lu-DOTATATE correlated with radiosensitivity, indicating that radiosensitisers may enhance the efficacy of ^177^Lu-DOTATATE treatment.

### Characterisation of the sensitivity of SK-N-BE(2c), CHLA-15 and CHLA-20 cells to treatment with nutlin-3 and topotecan alone or in combination

**Figure 2:**
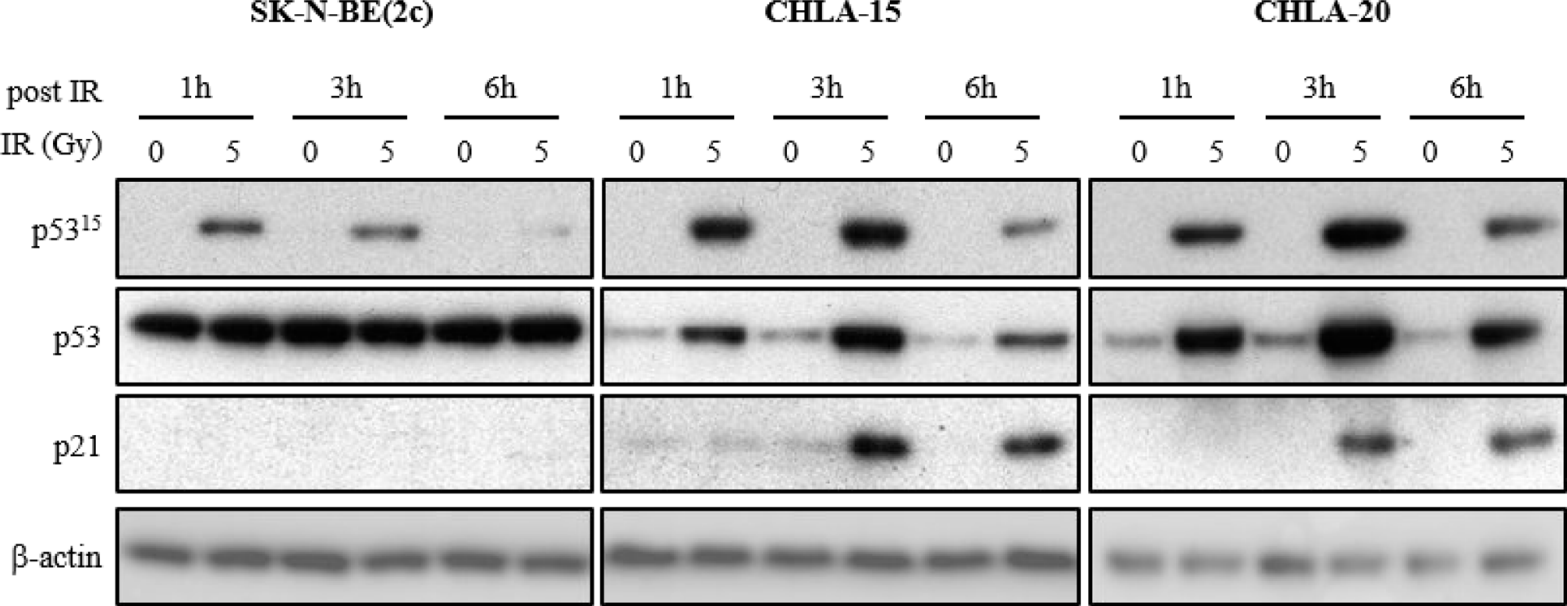
The effect of X-irradiation on p53 signalling in SK-N-BE(2c), CHLA-15 and CHLA-20 cells The expression of p53 phosphorylated at serine 15 (p53^15^), total p53 and p21 in SK-N-BE(2c), CHLA-15 and CHLA-20 cells was evaluated by immunoblotting 1, 3 and 6 h following X-irradiation with 5 Gy. p53^15^ is a marker of p53 activation by upstream kinases, p21 is a marker of p53 transcriptional activity and β-actin is used as a loading control.

In SK-N-BE(2c) cells, p53 expression was not increased in response to X-irradiation despite phosphorylation at serine 15 (Figure 2), a typical marker for p53 activation. The absence of p21^Cip1/Waf1^ (p21) expression indicated that the transcriptional activity of p53 was impaired at the p21 gene promoter in SK-N-BE(2c) cells in response to X-irradiation (Figure 2). This conclusion is supported by the previously reported detection, in SK-N-BE(2c) cells, of mutations in the DNA-binding domain of p53 [37]. In contrast, in CHLA-15 and CHLA-20 cells, the increase in p53 expression in response to X-irradiation was associated with its activation by phosphorylation at serine 15 and expression of p21 (Figure 2). This observation indicated that, in response to X-irradiation, p53 is an active transcription factor at the p21 gene promoter in CHLA-15 and CHLA-20 cells. Endogenous levels of p53 were higher in SK-N-BE(2c) cells than in CHLA-15 and CHLA-20 cells (Figure 2), perhaps reflecting a compensatory mechanism resulting from the impairment of p53 transcriptional activity.

**Figure 3:**
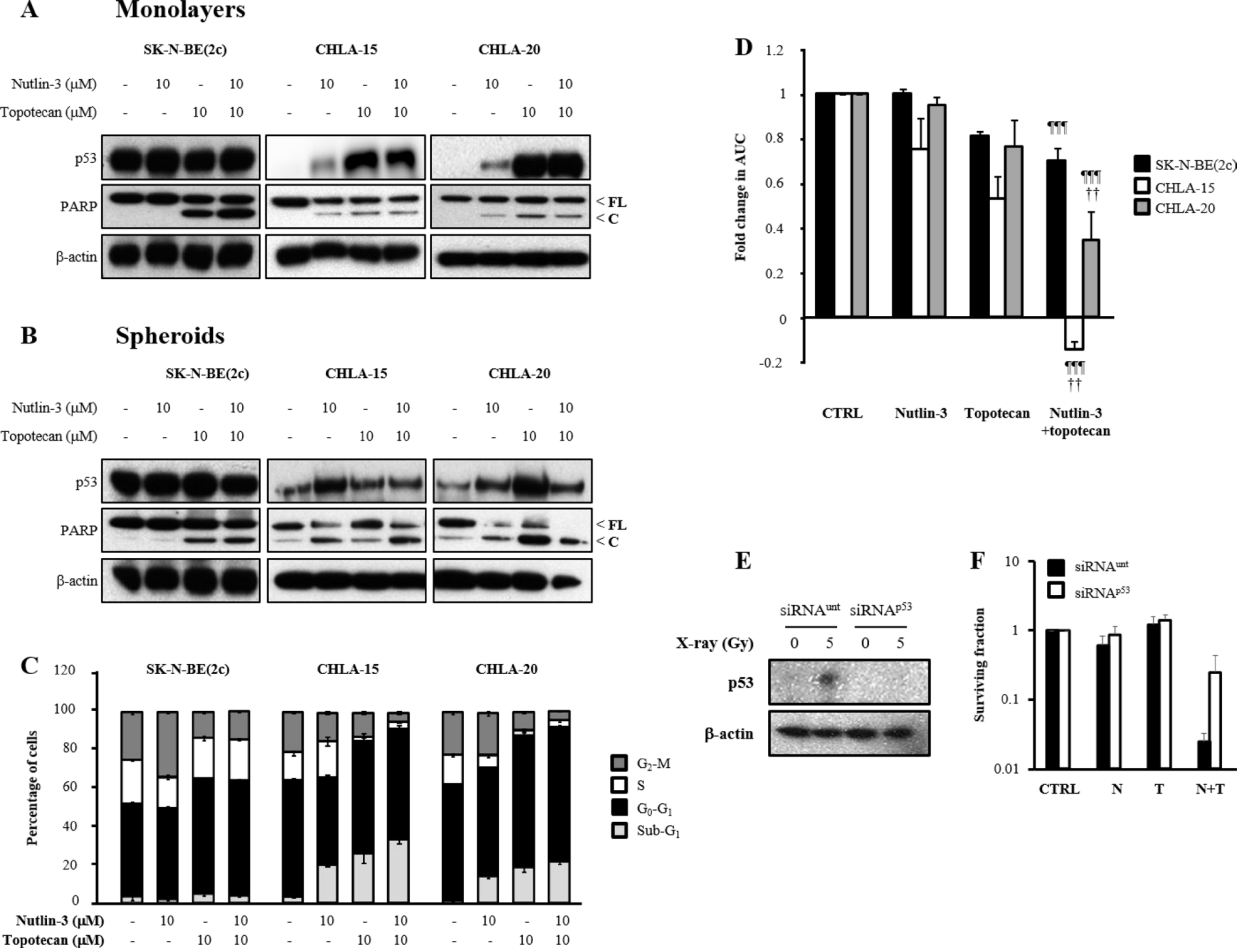
The effect of nutlin-3 and topotecan alone or in combination on the induction of p53 signalling, cell cycle arrest and spheroid growth delay The expression of p53, full-length (FL) and cleaved (C) PARP was evaluated by immunoblotting 6 h in monolayers (**A**) or 24 h in spheroids (**B**) following treatment of SK-N-BE(2c), CHLA-15 and CHLA-20 cells with nutlin-3 and topotecan alone or in combination. (**C**) The distribution of SK-N-BE(2c), CHLA-15 and CHLA-20 cells throughout the phases of the cell cycle was evaluated in monolayers by flow cytometry analysis of propidium iodide-stained cells following treatment for 12 h with nutlin-3 and topotecan alone or in combination. (**D**) SK-N-BE(2c), CHLA-15 and CHLA-20 spheroids were treated with 10 μM nutlin-3 and 10 μM topotecan alone or in combination for 24 h. The median AUC values resulting from each treatment were statistically compared with each other using Mann-Whitney pairwise comparisons. The symbol ^*^indicates a comparison with untreated control, the symbol ^¶^indicates a comparison with nutlin-3 alone and the symbol ^†^indicates a comparison with topotecan alone. One symbol indicates *P* < 0.05, two symbols indicate *P* < 0.01 and three symbols indicate *P* < 0.001. (**E**) Immunoblotting was used to determine the ability of p53-targeted siRNA (siRNA^p53^) or untargeted siRNA (siRNA^unt^) to abrogate the increase in p53 expression level following X-irradiation. SH-SY5Y cells were irradiated with 5 Gy and protein extracts harvested 3 h later. (**F**) SH-SY5Y cells exposed to either siRNA^unt^ or siRNA^p53^ were exposed to 200 nM nutlin-3 and 5 nM topotecan alone or in combination and the clonogenic cell kill was determined.

Next, we determined the effect of exposure of SK-N-BE(2c), CHLA-15 and CHLA-20 cells to nutlin-3 and topotecan on p53 expression and PARP cleavage, a marker of caspase activity which is a hallmark of apoptotic cell death. This analysis was carried out in cellular monolayers (Figure 3A) as well as in spheroids (Figure 3B). In SK-N-BE(2c) cells, combination treatment consisting of nutlin-3 with topotecan resulted in apoptotic cell death. This was probably due to topotecan alone since treatment with nutlin-3 as a single agent did not cause apoptosis (Figure 3A and 3B). In contrast, treatment of CHLA-15 and CHLA-20 cells with nutlin-3 and topotecan alone or in combination resulted in increased p53 expression and PARP cleavage (Figure 3A and 3B). Furthermore, an increase in BAX expression was observed following treatment of CHLA-20 cells with nutlin-3 and topotecan alone or in combination (data not shown). Together, these results indicated that treatment with nutlin-3 induced p53 expression and apoptotic cell death in CHLA-15 and CHLA-20 cells which harbour transcriptionally active p53, whereas topotecan induced apoptotic cell death irrespective of the p53 status of SK-N-BE(2c), CHLA-15 and CHLA-20 cells.

The effect of nutlin-3 and topotecan on cell cycle distribution was evaluated in SK-N-BE(2c), CHLA-15 and CHLA-20 cells grown as cellular monolayers (Figure 3C). The statistical analysis of the changes in the proportion of cells in Sub-G1, a measure of apoptosis induction, G0-G1, S and G2-M are summarised in Table 1. In SK-N-BE(2c) cells, nutlin-3 and topotecan alone or in combination did not cause a significant increase in sub-G1 population. This observation was contrasted by the significant increase in the sub-G1 population following nutlin-3 and topotecan alone or in combination in CHLA-15 and CHLA-20 cells (Figure 3C, Table 1). Topotecan caused a significant decrease in the proportion of cells in S and G2-M in CHLA-15 and CHLA-20 but not in SK-N-BE(2c) cells (Figure 3C, Table 1). The combination of nutlin-3 and topotecan induced, in CHLA-15 and CHLA-20 but not in SK-N-BE(2c) cells, a significant decrease in the G2-M population in comparison with the effect of nutlin-3 or topotecan as single agents (Figure 3C, Table 1). Together, these results indicated that, in the p53 functional cell lines CHLA-15 and CHLA-20, the depletion of cells in S and G2-M phases of the cell cycle by treatment with nutlin-3 and topotecan was probably due to apoptotic cell death in these particular phases of the cell cycle.

**Table 1:** Cell cycle analysis after treatment of cells with nutlin-3 and topotecan alone or in combination

SK-N-BE(2c)				
	Sub-G1	G0-G1	S	G2-M
Control	3.68 ± 1.64	46.6 ± 0.93	22.1 ± 0.25	23.58 ± 0.74
10 μM nutlin-3	2.44 ± 0.75	44.8 ±1.10	15.6 ± 1.24^*^	32.2 ± 0.56^***^
10 μM topotecan	5.31 ± 0.98	58.6 ± 0.52^*^	20.6 ± 1.19	13.1 ± 0.50^***^
Nutlin-3 + toptecan	4.02 ± 0.17	58.5 ± 0.85^¶¶¶^	21.0 ± 0.53^¶¶^	14.2 ± 0.29^¶¶¶^

CHLA-15				
	Sub-G1	G0-G1	S	G2-M
Control	3.16 ± 0.60	58.8 ± 3.67	14.3 ± 2.81	20.6 ± 1.13
10 μM nutlin-3	19.8 ± 1.70^**^	44.7 ± 3.65^*^	18.8 ± 5.34	14.6 ± 1.60^***^
10 μM topotecan	25.8 ± 12.2^***^	58.2 ± 10.6	1.73 ± 0.33^***^	12.8 ± 1.90^***^
Nutlin-3 + toptecan	33.1 ± 5.36^¶^	56.9 ± 5.04^¶^	3.35 ± 0.59^¶¶¶^	4.57 ± 0.74^¶¶¶†††^

CHLA-20				
	Sub-G1	G0-G1	S	G2-M
Control	1.73 ± 0.26	59.7 ± 0.76	15.6 ± 0.67	21.6 ± 0.84
10 μM nutlin-3	14.4 ± 1.23^***^	56.5 ± 0.81	6.54 ± 0.53^***^	21.9 ± 1.15
10 μM topotecan	18.8 ± 1.99^***^	68.7 ± 1.78^***^	2.78 ± 0.63^***^	9.41 ± 0.51^***^
Nutlin-3 + toptecan	22.2 ± 1.40^¶¶^	69.3 ± 1.05^¶¶¶^	3.49 ± 0.65^¶^	4.79 ± 0.20^¶¶¶††^

Propidium iodide-stained cells were analyzed using flow cytometry to determine cell cycle distribution 24 h following treatment of SK-N-BE(2c), CHLA-15 and CHLA-20 cells with 10 μM nutlin-3 and topotecan alone or in combination. One-way ANOVA with Bonferroni correction was performed. ^*^, indicates a comparison with untreated control, ^¶^indicates a comparison with nutlin-3 alone, ^†^indicates a comparison with topotecan alone. One symbol indicates *P* < 0.05, two symbols indicate *P* < 0.01 and three symbols indicate *P* < 0.001.

Finally, as spheroids grow, their heterogeneous internal morphology is characterised by a hypoxic, non-dividing and dying core encircled by a proliferating outer layer [23]. The growth delay experiments were designed to treat the spheroids when they were 100 μm in diameter and to monitor them until they grew to a diameter of 1 μm. The exposure of 100 μm diameter spheroids to the combination treatment consisting of nutlin-3 with topotecan resulted in a significantly increased growth delay in CHLA-15 and CHLA-20 spheroids in comparison with single agent treatments (Figure 3D, Supplementary Figure 2). In contrast, there was no significant enhancement of topotecan-induced growth delay in SK-N-BE(2c) spheroids by combination with nutlin-3 (Figure 3D).

Together, these results suggested that the combination treatment consisting of nutlin-3 and topotecan may be more potent in tumours capable of activating p53 signalling. To evaluate this conjecture, we performed p53 gene expression knockdown in SH-SY5Y cells followed by evaluation of the clonogenic cell kill induced by the combination of nutlin-3 and topotecan. We hypothesised that transfection of SK-N-BE(2c) cells with a p53-expressing vector would not result in a functional p53 axis due to the endogenous expression of mutant p53. In response to X-irradiation, there was an increase in p53 expression in SH-SY5Y cells exposed to untargeted siRNA but not in SH-SY5Y cells treated with p53-targeted siRNA (Figure 3E). In response to combination treatment consisting of nutlin-3 with topotecan, there was a 10-fold increase in clonogenic cell growth in p53-silenced SH-SY5Y cells in comparison with SH-SY5Y cells exposed to untargeted siRNA (Figure 3F). This observation indicated that cytotoxicity resulting from the combination of nutlin-3 with topotecan depends on a functional p53 signalling. In agreement, it has previously been reported that nutlin-3 and topotecan interact in a synergistic manner in cell lines with wild-type p53 [30, 31].

**Figure 4:**
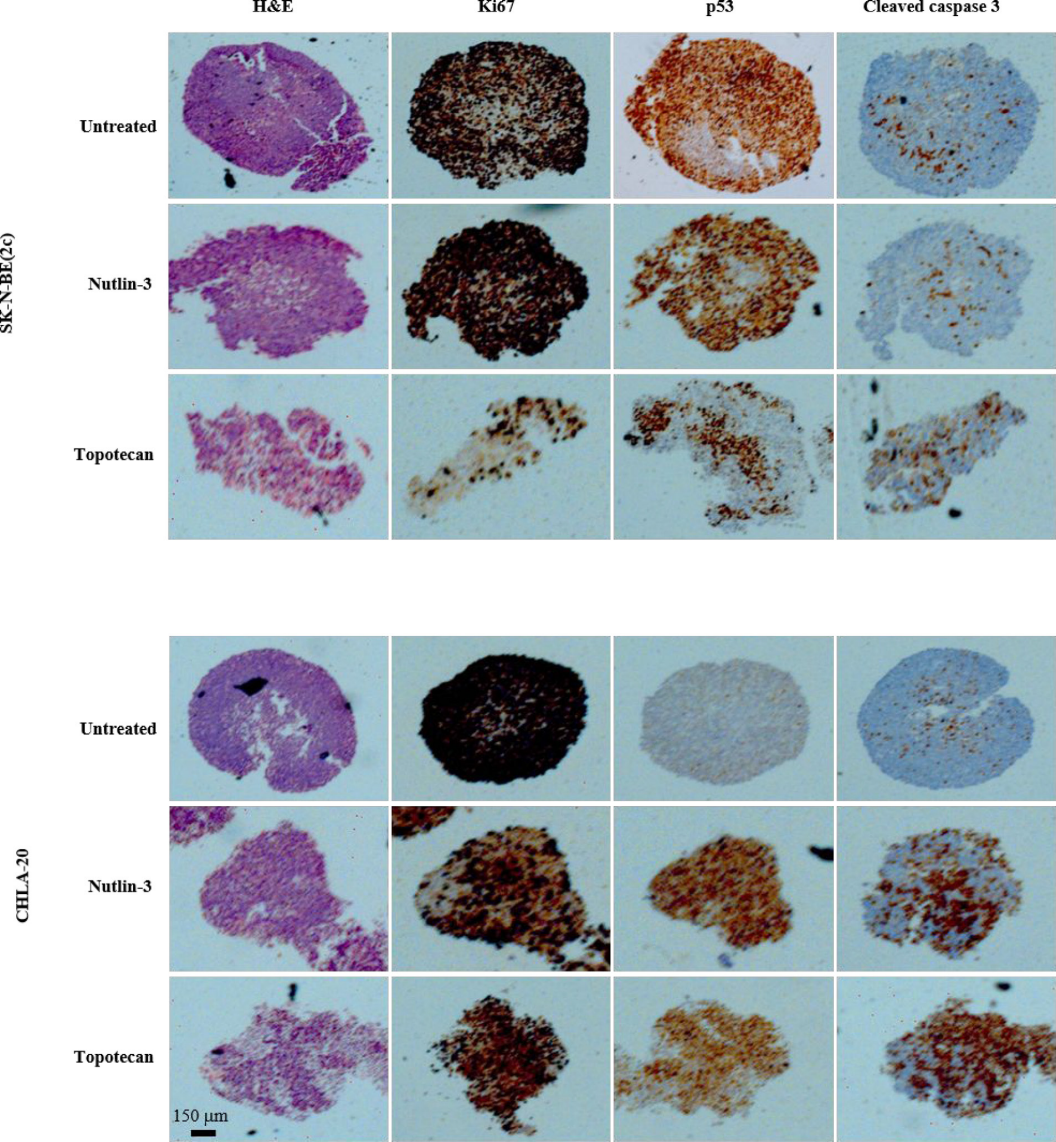
The effect of nutlin-3 and topotecan on cellular proliferation, p53 expression and apoptotic cell death in SK-N-BE(2c) and CHLA-20 spheroids The markers of histological organisation (haematoxylin and eosin, H&E), proliferation (Ki-67), p53 expression and apoptotic cell death (cleaved caspase 3) were detected by immunohistochemistry in SK-N-BE(2c) and CHLA-20 spheroids. The spheroids were allowed to grow for 3 weeks to reach 1 mm in diameter prior exposure to 10 μM nutlin-3 or 10 μM topotecan for 24 h.

The sensitivity of tumours to cytotoxic treatment is known to depend on their size which influences drug penetration and resistance to treatment due to heterogeneous areas of quiescence and/or hypoxia [38]. Accordingly, we evaluated the effect of spheroid size on the cytotoxicity of nutlin-3 and topotecan. SK-N-BE(2c) and CHLA-20 spheroids were allowed to grow for 3 weeks before exposure to nutlin-3 and topotecan as single agents. CHLA-15 spheroids were not recovered because of extensive disaggregation caused by the treatments. Similarly, combination treatments of SK-N-BE(2c) and CHLA-20 spheroids resulted in disaggregation and loss of spheroid morphology which precluded harvesting for immunohistochemistry. In CHLA-20, but not in SK-N-BE(2c) spheroids, nutlin-3 and topotecan treatments increased expression of p53 and cleaved caspase 3, a marker of apoptotic cell death (Figure 4). In SK-N-BE(2c) spheroids, only topotecan treatment increased expression of cleaved caspase 3 (Figure 4). Importantly, caspase 3 cleavage was observed throughout the spheroid, indicating that the phenomenon of drug resistance due to poor tissue penetration is unlikely in micrometastases. Furthermore, the induction, by nutlin-3 and topotecan, of apoptotic cell death throughout spheroids, suggested that, as well as targeted radiopharmaceuticals, nutlin-3 and topotecan are suitable treatment modalities for malignant lesions in the class size of micrometastases.

### Enhancement of ^177^Lu-DOTATATE-induced spheroid growth delay by nutlin-3 and topotecan

Both topotecan and nutlin-3 have been shown to enhance targeted radiotherapy and to be radiosensitisers [12, 23, 24]. Therefore we evaluated the effect of nutlin-3 and topotecan alone or in combination on the spheroid growth delay induced by ^177^Lu-DOTATATE. The combination of 10 μM of nutlin-3 and topotecan sterilised CHLA-15 spheroids (Figure 3D, Supplementary Figure 2). Consequently, in order to observe modulation of the spheroid growth delay by combination with ^177^Lu-DOTATATE, CHLA-15 spheroids were also treated with an alternative, lower dosage of nutlin-3 and topotecan. Combined treatment with 5 μM nutlin-3 and 5 μM topotecan resulted in a significantly increased growth delay (*P* < 0.01) in CHLA-15 spheroids in comparison with single agent treatments, without sterilisation (Supplementary Figure 3).

**Figure 5:**
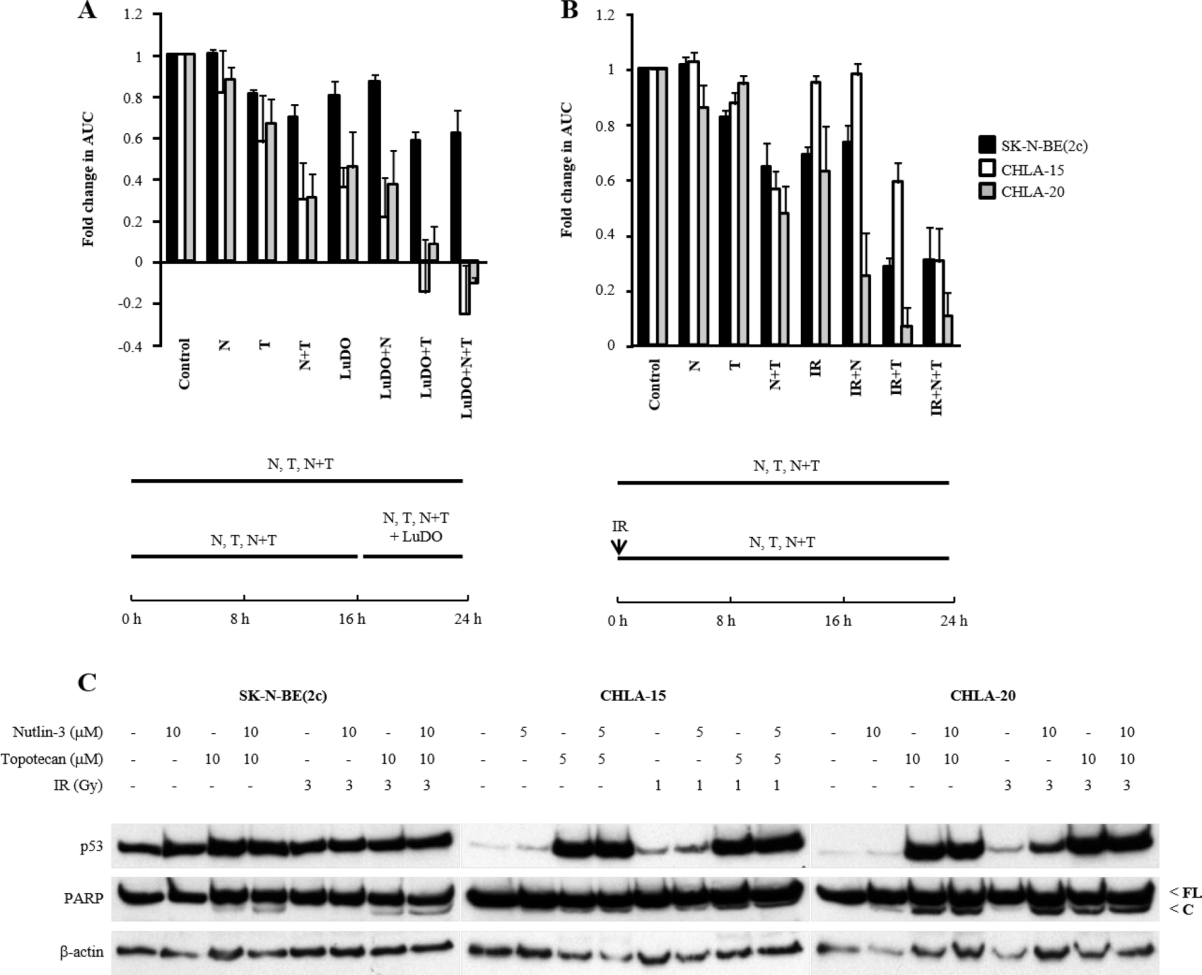
The effect of nutlin-3 and topotecan alone or in combination on the spheroid growth delay induced by X-irradiation or ^177^Lu-DOTATATE (**A**) SK-N-BE(2c) and CHLA-20 spheroids were treated with 10 μM nutlin-3 (N) and 10 μM topotecan (T) for 24 h. CHLA-15 spheroids were treated with 5 μM nutlin-3 and 5 μM topotecan. ^177^Lu-DOTATATE (LuDO) treatment consisted of exposure for 8 h to 5 MBq/ml. The schedule of administrations of nutlin-3, topotecan and ^177^Lu-DOTATATE is shown below the histogram. (**B**) SK-N-BE(2c), CHLA-15 and CHLA-20 spheroids were pre-treated with nutlin-3 and topotecan alone or in combination for 24 h. The spheroids were irradiated (IR) with 3 Gy, 1 Gy and 3 Gy, respectively, at the start of exposure to nutlin-3 and topotecan. The schedule of administrations of nutlin-3, topotecan and IR is shown below the histogram. (**C**) The expression of p53, full-length (FL) and cleaved (C) PARP was evaluated by immunoblotting 6 h following treatment of SK-N-BE(2c), CHLA-15 and CHLA-20 monolayers with nutlin-3, topotecan and X-irradiation alone or in combination. (A, B) The median AUC values resulting from each treatment were statistically compared with each other using Mann–Whitney pairwise comparisons. However, statistical significance was not reached.

Firstly, we determined the spheroid growth delay resulting from treatment with nutlin-3 and topotecan alone or in combination with ^177^Lu-DOTATATE. Although not statistically significant, topotecan and the combination treatment consisting of nutlin-3 with topotecan enhanced the spheroid growth delay induced by ^177^Lu-DOTATATE (Figure 5A, Supplementary Figure 4A). Moreover, inhibition of proliferation was more prominent in CHLA-15 and CHLA-20 spheroids, which harbour a functional p53 signalling pathway, than in SK-N-BE(2c) spheroids which do not (Figure 5A, Supplementary Figure 4A).

Secondly, the effect of nutlin-3 and topotecan alone or in combination on X-irradiation-induced spheroid growth delay was evaluated to determine whether radiosensitisation could explain the enhancement of ^177^Lu-DOTATATE efficacy. Similarly to the combination treatments with ^177^Lu-DOTATATE, topotecan and the combination treatment consisting of nutlin-3 with topotecan enhanced the spheroid growth delay induced by X-irradiation although statistical significance was not reached (Figure 5B, Supplementary Figure 4B). Furthermore, growth inhibition was more prominent in CHLA-15 and CHLA-20 than in SK-N-BE(2c) spheroids. For instance, nutlin-3 enhanced X-irradiation-induced spheroid growth delay to a greater extent in CHLA-20 spheroids than in SK-N-BE(2c) spheroids (Figure 5B). This observation was consistent with the enhanced increase in p53 and cleaved PARP expression following combination treatment consisting of nutlin-3 and X-irradiation in comparison with nutlin-3 alone or X-irradiation alone in CHLA-20 cells but not in SK-N-BE(2c) cells (Figure 5C). Similarly, the triple combination consisting of nutlin-3, topotecan and X-irradiation increased growth delay in CHLA-15 spheroids to a greater extent than in SK-N-BE(2c) spheroids (Figure 5B). PARP cleavage and p53 expression may already be maximal after treatment with topotecan and no further increase was possible following triple combination treatment consisting of nutlin-3, topotecan and X-irradiation (Figure 5C).

## DISCUSSION

Multicellular tumour spheroids are well-established models of prevascular micrometastases that provide a means of studying the intratumoural distribution of therapeutic agents and of determining the effect of cytotoxic agents. They have previously been used extensively in targeted therapy research to investigate diffusion gradients of targeting agents, to assess efficacies of alternative modalities, to evaluate microdosimetry and to provide experimental model systems for testing hypotheses [11]. In this study, using multicellular tumour spheroids, we show that the radiosensitising combination consisting of nutlin-3 and topotecan enhanced the spheroid growth delay induced by ^177^Lu-DOTATATE. This effect was more prominent in spheroids composed of cells which harbour functional p53 signalling and constitutes an appropriate therapeutic option to target malignant lesions the size of micrometastases.

Due to the cytotoxic and cytostatic effects of somatostatin, the expression of SSTRs has been found to inversely correlate with MYCN amplification and to positively correlate with survival of patients with differentiated neuroblastomas [39–41]. However, these studies also found a subset of neuroblastoma tumours with MYCN amplification or classified as stage III-IV that were characterised by levels of SSTRs expression equivalent to tumours with a better prognosis [39–41]. Therefore, ^177^Lu-DOTATATE therapy is suitable for high risk neuroblastoma in a subset of patients with a positive ^68^Ga-DOTATATE diagnostic scan.

We found that SSTR2 expression levels were greater in SK-N-BE(2c) than in CHLA-15 and CHLA-20 cells (Figure 1C). This result was corroborated by the significant increased uptake of ^177^Lu-DOTATATE by SK-N-BE(2c) cells than by CHLA-15 and CHLA-20 cells (Figure 1B). Although, it has recently been reported that SK-N-BE(2c) cells express lower levels of SSTR2 than CHLA-15 and CHLA-20 cells, this study did not report the outcome of a functional assay [42]. Furthermore, in our study, we observed that the spheroid growth delay induced by ^177^Lu-DOTATATE increased with increasing duration of exposure. This observation might be explained by the intracellular trafficking of SSTRs following agonist-induced internalisation [43]. For instance, it has been shown that SSTR2 is transported intracellularly and recycled back to the membrane within 1 h following octreotide stimulation [44]. The recycling of SSTR2 back to the membrane within 1h could allow the binding and internalisation of a second ^177^Lu-DOTATATE molecule. Thus, the longer the exposure to ^177^Lu-DOTATATE, the greater the internalised amount of ^177^Lu-DOTATATE. In conjunction with the prolonged retention of ^177^Lu-DOTATATE within tumours [45], the time-dependent cytotoxicity of ^177^Lu-DOTATATE can be seen as a positive aspect of this therapy.

Recently, microtubule polymerisation has been suggested to be a target of topotecan, thereby challenging the opinion that topotecan’s cytotoxicity is due solely to topoisomerase I inhibition [46]. In our study, we found that 10 μM topotecan as a single agent specifically killed cells in S and G2-M phases of the cell cycle (Table 1). However, it has been reported that this concentration may be too high to inhibit microtubule polymerisation [47]. Therefore, it is expected that the cellular depletion in the G2-M phase of the cell cycle is due to S phase-specific cell death rather than to interference with microtubule polymerisation during mitosis. Since S phase cells are radioresistant, radiosensitisation by topotecan may be explained by S phase-specific cell death. Furthermore, the combination of nutlin-3 with topotecan caused a significant decrease in the proportion of cells in G2-M (Table 1). The depletion of cells in S phase by topotecan alone and the depletion of cells in G2-M by the combination of nutlin-3 with topotecan were only significant in CHLA-15 and CHLA-20 cells - which are capable of activating p53 signalling - but not in SK-N-BE(2c) cells. This observation can be explained by the known roles of p53 in cell death and the activation of cell cycle checkpoints in S and G2 [47].

It has been shown that the p53 status of a tumour cell is a determinant of sensitivity to topotecan although this is subject to debate. For instance, it has been reported that p53 triggers the proteasomal degradation of topoisomerase I leading to resistance to topotecan [48] and that topotecan treatment is more potent in p53-deficient cells because of downregulation of XIAP and survivin [49]. In contrast, it has been suggested that the p53 status of glioma cell lines does not predict sensitivity to topotecan [50]. Importantly, in SK-N-BE(2c) spheroids, unable to activate p53 signalling, treatment with nutlin-3 in combination with topotecan did not reverse the enhancement of spheroid growth delay induced by topotecan. This observation suggests that although the combination of MDM2 inhibitors and topotecan is likely to provide more benefit for patients whose tumours are p53 functional, this combination treatment may not result in a lower efficacy in patients with p53 non-functional tumours. Therefore, if MDM2 inhibitors are found to be clinically safe, these molecules may be given in addition to topotecan regardless to the p53 status of the patient’s tumour, thereby eliminating the need for the development of biomarkers of p53 signalling.

The selection of patients who will most likely benefit from therapy is a major area of medical research. The levels of MDM2 expression have been reported to be prognostic and predictive biomarkers [51, 52]. Specifically, MDM2 expression or the presence of the MDM2 SNP309 polymorphism, which causes increased expression of the MDM2 gene, correlated negatively with outcome in patients with mesothelioma [53, 54]. Furthermore, MDM2 expression or the presence of the MDM2 SNP309 genetic variant correlated positively with sensitivity to chemotherapeutic agents including topoisomerase I inhibitors [53]. These studies suggest that MDM2 amplification and the MDM2 polymorphism SNP309 have prognostic value and may serve as predictive biomarkers of chemotherapy efficacy.

Recently, p53-independent MDM2 functions have been described with respect to possible adverse side-effects in patients [54]. Specifically, MDM2 binds to the retinoblastoma tumour suppressor (Rb) and promotes its degradation [54]. Besides its tumour suppressor activity, Rb also regulates muscle development and myoblast proliferation and differentiation [54]. It has been shown that nutlin-3 treatment, which results in increased MDM2 levels due to p53 activation, leads to Rb degradation and decreased levels of myoblast proliferation and differentiation markers, suggesting potential clinical complications [54]. However, the strategy consisting of reactivating p53 is currently being tested in various phase I clinical trials of cancer in order to assess safety and tolerability [55]. For instance, the inhibitor of the p53-MDM2 interaction, MK-8242, was investigated in 47 patients with p53-wild-type solid tumours [56]. Its safety and tolerability at dosage producing clinical responses provided a rationale for further testing in a phase II clinical trial [56]. In another study, responses have been observed following administrations of MDM2 inhibitors with acceptable toxicity profiles [57]. Our present study, and others [30–33], suggest that the optimal use of nutlin-3 may be in combination with alternative therapies. Since ^177^Lu-DOTATATE concentrates in malignant lesions, the enhancement of chemotherapy efficacy by the combination with nutlin-3 only occurs in targeted cells, thus sparing normal tissues and reducing the gravity of adverse effects.

The goal of combination therapy is the potentiation of tumour-specific cytotoxicity at lower dosages of therapeutic agents while avoiding an increase in the risk of adverse effects in healthy tissues. The blending of drugs with non-overlapping mechanisms of action that function in concert to reactivate p53 may be a strategy to achieve this goal. Firstly, MDMX is structurally related to MDM2 and reduces p53 activity [18, 19]. The strategy consisting of dual inhibition of the p53-MDM2 and p53-MDMX interactions has shown promise in pre-clinical testing [58]. Secondly, the p53 gene product Wip1/PPM1D is a phosphatase which dephosphorylates and deactivates p53 [59]. The combination of nutlin-3 with Wip1 inhibition has been shown to be synergistic and to potently reactivate p53 signalling [60]. Interestingly, it has been shown that the novel Wip1 inhibitor GSK2830371 reduced the growth of neuroblastoma xenografts in mice and enhanced the cytotoxicity caused by topoisomerase II inhibitors [61]. In the light of these observations, the radiosensitising properties of the nutlin-3 and topotecan combination may be safely enhanced further by dual inhibition of p53-MDM2 and p53-MDMX interactions or by inhibition of Wip1.

In summary, this study indicates that the efficacy of ^177^Lu-DOTATATE treatment of children with high risk neuroblastoma may be enhanced by the combination with the radiosensitising drugs nutlin-3 and topotecan. The precise dose-scheduling which will be optimal remains to be determined. Interestingly, it was recently reported that a single injection of ^177^Lu-DOTATATE in mice bearing CHLA-15 xenografts did not induce a significant long-lasting growth delay [42], indicating that combination of ^177^Lu-DOTATATE with radiosensitisers may be beneficial. Further experiments in animal models of neuroblastoma are therefore warranted to facilitate the clinical development of the combination of nutlin-3 and topotecan with ^177^Lu-DOTATATE. This strategy holds promise as another component in the multimodality arsenal for the treatment of children with high risk neuroblastoma.

## MATERIALS AND METHODS

### Cell culture

The neuroblastoma cell lines CHLA-90, CHLA-15 and CHLA-20 were obtained from the Children’s Oncology Group and maintained in Iscove’s Modified Dulbecco’s medium supplemented with 2 mM L-glutamine, 1% (v/v) insulin, 1% (v/v) transferrin, 1% (v/v) selenium and 20% (v/v) foetal bovine serum (FBS, Autogen Bioclear, UK) at 37° C in a 5% CO_2_ atmosphere. The neuroblastoma cell lines SK-N-BE(2c), SK-N-SH and SH-SY-5Y and the phaeochromocytoma cell line PC12 were obtained from the American Tissue Culture Collection. SK-N-BE(2c) cells were maintained in Dulbecco’s Modified Eagle medium (DMEM) supplemented with 15% (v/v) FBS, 2 mM L-glutamine and 1% (v/v) non-essential amino acids at 37° C in a 5% CO_2_ atmosphere. PC12, SH-SY5Y and SK-N-SH cells were maintained in DMEM medium supplemented with 10% (v/v) FBS and 2 mM L-glutamine at 37° C in a 5% CO_2_ atmosphere. The glioma cell line UVW was described previously [62]. UVW cells were maintained in Minimum Essential Medium supplemented with 10% (v/v) FBS and 2 mM L-glutamine at 37° C in a 5% CO_2_ atmosphere. Unless otherwise stated, all reagents used for cell culture were purchased from ThermoFisher Scientific (UK).

### Silencing of p53 expression

The transfection of siRNAs was performed with Lullaby^®^ siRNA transfection reagent according to the manufacturer’s protocol (OZ Biosciences, Marseille, France). The sense sequence of the p53-targeted siRNA was GAC UCC AGU GGU AAU CUA CUU Dharmacon (Lafayette, Colorado). The siGENOME non-targeting siRNA pool #2 from Dharmacon (Lafayette, Colorado) was used as a negative control. Following exposure to 124 nM siRNAs for 72 h, the cells were suspended by treatment with trypsin and seeded in triplicates at 2,000 cells per well in 6-well plates. Once attached, the cells were exposed to 200 nM nutlin-3 and 5 nM topotecan alone and in combination and incubated at 37° C in 5% CO_2_ to allow for colony formation. Once the colonies comprised more than 50 cells, the medium was removed and the colonies were fixed in 50% methanol (v/v) in PBS and stained with 1% (v/v) crystal violet in PBS before counting.

### ^177^Lu-DOTATATE uptake assay

^177^Lu-DOTATATE was obtained from Advanced Accelerator Applications, France. The specific activity was 37 GBq/mg. Cellular monolayers were incubated for 2, 4, 6 and 8 h at 37° C in a 5% CO_2_ atmosphere in culture medium containing 100 kBq/ml ^177^Lu-DOTATATE in the presence or in the absence of 1 μM octreotide, a competitive inhibitor of binding to SSTR. The concentration of octreotide and the radioactivity concentration of ^177^Lu-DOTATATE were selected based on our preliminary investigation (data not shown). They were then washed three times in PBS. The radioactivity retained in the cells was extracted with 10% (w/v) trichloroacetic acid and measured using a γ-counter (Canberra Packard, UK). The number of cells was counted using a haemocytometer in a separate identical culture and ^177^Lu-DOTATATE uptake was expressed as counts per minute (cpm) per cell.

### Spheroid initiation

Spheroids were obtained using the liquid overlay technique [63]. Cellular monolayers were trypsinised and reseeded at a cellular density of 160,000 cells.cm^-2^ in ultra-low attachment flasks (Corning, Netherlands). Spheroids formed within 2 days.

### Treatment of spheroids and growth curve analysis

In order to assess the efficacy of radiopharmaceuticals combined with radiosensitisers, we selected the spheroid growth delay. A three-dimensional culture system is required to adequately represent therapeutic effects upon micrometastases (a major aim of targeted radionuclides) and because radionuclides are toxic to tumours not only because their decay particles inflict damage in targeted cells but also because cross-fire irradiation to neighbouring, untargeted cells contributes significantly to efficacy. Finally, spheroid growth was monitored for 21 days - a period long enough to observe at least a 100-fold increase in volume. Twenty-one days is similar to the time period required for the formation of colonies in clonogenic assay. Single agent treatments consisted of exposure of the spheroids to ^177^Lu-DOTATATE for 8 h or to nutlin-3 and topotecan for 24 h. Combination treatments consisted of exposure of the spheroids to nutlin-3 and/or topotecan for 16 h followed by exposure to nutlin-3 and/or topotecan in combination with ^177^Lu-DOTATATE for 8 h. At the end of treatments, the spheroids were washed three times in PBS. Then, those of approximately 100 μm in diameter were manually selected and individually transferred into ultra-low attachment plates for monitoring (Corning, Netherlands). For every spheroid, two orthogonal diameters, d_max_ and d_min_, were measured twice per week using the image analysis software ImageJ (National Institute of Health, Bethesda, MD, USA) and the volume, V, was calculated using: V = π × d_max_ × d_min_^2^/6,000,000 [64]. The spheroid volume, at various time points after the initiation of treatment, was computed as the quotient of volume at time t divided by volume at time zero (V/V_0_). In order to evaluate the effect of treatment over the course of an experiment, the area under the log V/V_0_ versus time curve (AUC) was calculated for individual spheroids using trapezoidal approximation. Spheroid growth delay caused by a treatment was indicated by a reduction of the AUC value in comparison with that of the untreated spheroids.

### X-ray irradiation

Monolayers or spheroids were irradiated using an RS225 irradiator (Xstrahl, UK) at room temperature with 195 kV/10 mA X-rays producing a dose rate of 1.64 Gy/min at a distance of 30 cm.

### Statistical analysis of spheroid growth delay

The distribution of AUC values was not normal, as indicated by the Shapiro–Wilk test. Therefore, non-parametric Kruskal–Wallis testing was used to determine whether experimental data indicated a significant level of difference between the medians of the groups. If the *P*-value corresponding to the Kruskal–Wallis test was less than 0.05, the Mann–Whitney test was used for pairwise comparisons. The comparison of single agent treatment with combination treatment was characterised by the following features. Firstly, in order to demonstrate enhancement of ^177^Lu-DOTATATE-induced spheroid growth delay, the observed effect in response to combination treatment of a radiosensitiser with ^177^Lu-DOTATATE had to be greater than that induced by ^177^Lu-DOTATATE or the radiosensitiser alone. Secondly, an absence of enhancement of ^177^Lu-DOTATATE-induced growth delay could be due to insufficient radiosensitiser dosage. Therefore, the evaluation of the modification of the effect of ^177^Lu-DOTATATE involved a family of four pairwise comparisons: radiosensitiser versus untreated control, ^177^Lu-DOTATATE versus untreated control, radiosensitiser + ^177^Lu-DOTATATE versus radiosensitiser and radiosensitiser + ^177^Lu-DOTATATE versus ^177^Lu-DOTATATE. To compensate for multiple pairwise comparisons, Bonferroni correction was applied. In order to retain the criterion *P* < 0.05, the level of significance of each pairwise comparison was set to 0.0125.

### Immunoblotting

Antibodies against SSTR2 and β-actin were obtained from Abcam, UK. The antibody against p53 was obtained from Santa-Cruz, US. Antibodies against p53 phosphorylated at serine 15 and PARP were obtained from New England Biolabs, UK. The antibody against p21 was obtained from BD Pharmingen, UK. Whole cellular protein extracts were resolved in reducing and denaturing conditions by sodium dodecyl sulphate polyacrylamide gel electrophoresis. Proteins were transferred on to polyvinylidene fluoride (PVDF) Immobilon-P membranes (Millipore, UK). Membranes were blocked with 7.5% (w/v) milk for 2 h prior to incubation with the primary antibodies overnight at 4° C. Membranes were then washed and incubated at room temperature for 1 h with horseradish peroxidase-conjugated secondary anti-mouse (Cell Signalling, UK) or anti-rabbit antibody (Cell Signalling, UK) to enable chemiluminescent detection using ECL (ThermoFisher Scientific, UK).

### Cell cycle analysis

SK-N-BE(2c), CHLA-15 and CHLA-20 cellular monolayers were exposed for 12 h to 10 μM nutlin-3 and 10 μM topotecan alone or in combination. After 12 h, the cells were harvested by trypsinisation and fixed in 70% (v/v) ethanol at −20° C. The cells were stained with 10 μg/ml propidium iodide and 200 μg/ml RNAse A for at least 10 min prior to analysis using a FACSCalibur flow cytometry system (BD Biosciences, UK). Flow cytometric data were quantified using FlowJo 7.6.5 software.

### Immunocytochemistry

After treatment, the spheroids were fixed in 10% neutral formalin (Cellpath, UK) for 24 h at 4° C. The spheroids were agitated and gently mixed before being carefully placed at the centre of biopsy paper (Leica, UK). The biopsy paper was then folded over twice and placed into the tissue processing cassette for processing through 70, 90, 95% (v/v) alcohol, 100% (v/v) ethanol, xylene and molten wax. The solid wax that may have formed was removed by placing the unfolded biopsy paper containing the spheroids onto a hot plate. Using heated forceps, the spheroids were then transferred from the biopsy paper into a tissue embedding mold containing molten wax. A tissue cassette was finally placed onto the embedding mold and left to harden. Once hardened, the paraffin wax block was removed and 4 μm sections were obtained using a Finesse microtome (ThermoFischer, UK). Spheroid sections were de-waxed in xylene and re-hydrated by successive immersions in graded alcohol and tap water. Endogenous peroxidase activity was quenched using peroxidase-blocking solution (Dako, UK). Heat-induced antigen retrieval was performed in a 10 mM sodium citrate, 0.05% (v/v) Tween 20, pH6 buffer at 98° C for 25 minutes using a pre-treatment module (Dako, UK). The sections were then washed using Tris-buffered Tween before being exposed to anti-Ki67 (ThermoFisher Scientific, UK), anti-p53 (Leika, UK) or anti-cleaved caspase 3 (Cell Signalling, UK) antibodies. Species appropriate secondary antibodies (Dako EnVision, UK) were applied for 30 min followed by 3,3′-diaminobenzidine tetrahydrochloride for 10 min. The reaction was terminated with deionized water for 1 min. The sections were then counterstained with Gills haematoxylin and the nuclei blued using Scott’s tap water. The sections were then dehydrated through graded alcohol, cleared in xylene and mounted with a glass coverslip using DPX mountant for microscopy.

## SUPPLEMENTARY MATERIALS FIGURES


