# Supplementary material for: An evaluation in vitro of the efficacy of nutlin-3 and topotecan in combination with 177Lu-DOTATATE for the treatment of neuroblastoma
Source: Oncotarget. 2018 Jun 26;9(49):29082–96. doi: 10.18632/oncotarget.25607 (PMC6044389; doi:10.18632/oncotarget.25607)
Supplement: Supplementary file 1 [file oncotarget-09-29082-s001.pdf]

An evaluation *in vitro* of the efficacy of nutlin-3 and topotecan in combination with ^{177}Lu-DOTATATE for the treatment of neuroblastoma

SUPPLEMENTARY MATERIALS

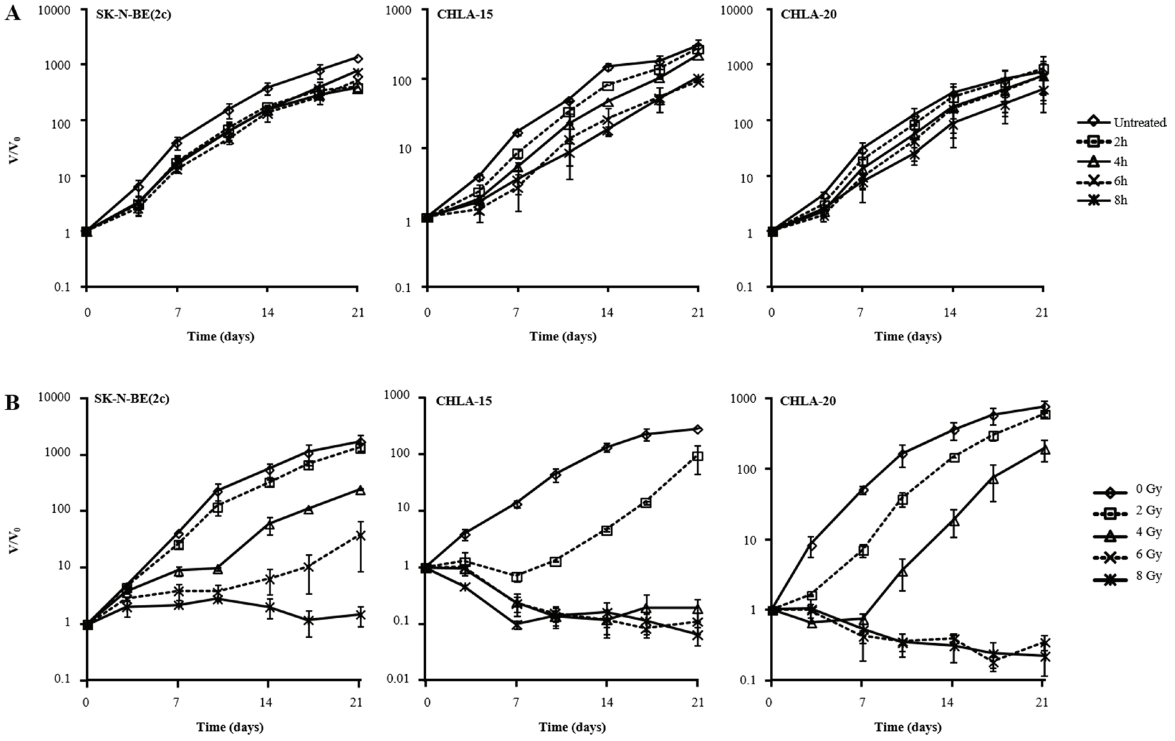

Supplementary Figure 1: The spheroid growth curves resulting from exposure to ^{177}Lu-DOTATATE or from X-irradiation. SK-N-BE(2c), CHLA-15 and CHLA-20 spheroids were exposed to 5 MBq/ml ^{177}Lu-DOTATATE for 2, 4, 6 or 8 h (**A**) or were X-irradiated with 2, 4, 6 or 8 Gy (**B**). The volume, at time t, was calculated as described in Materials and Methods then divided by their initial volume at t_0 to obtain the fold increase in volume (V/V_0).

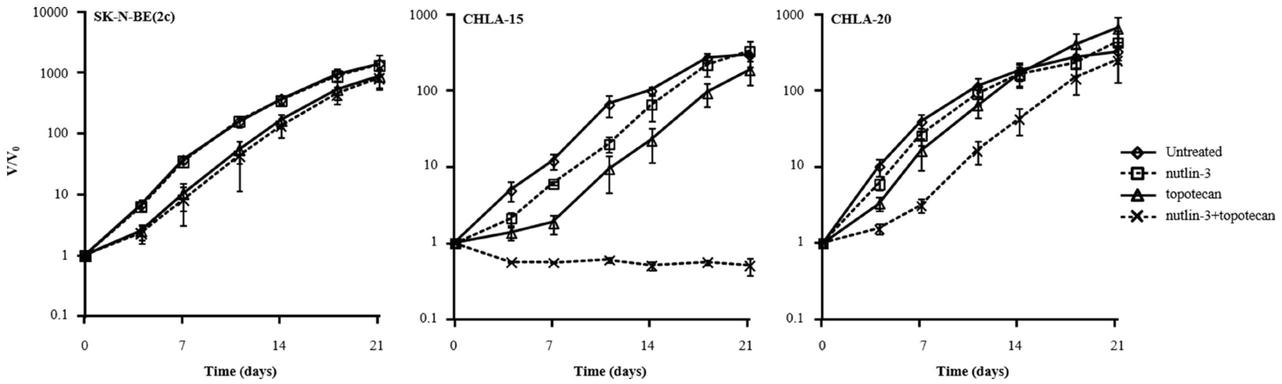

Supplementary Figure 2: The spheroid growth curves resulting from treatment with nutlin-3 and topotecan alone or in combination. SK-N-BE(2c), CHLA-15 and CHLA-20 spheroids were treated with 10 µM nutlin-3 and 10 µM topotecan alone or in combination for 24 h. The volume, at time t, was calculated as described in Materials and Methods then divided by their initial volume at t_0 to obtain the fold increase in volume (V/V_0).

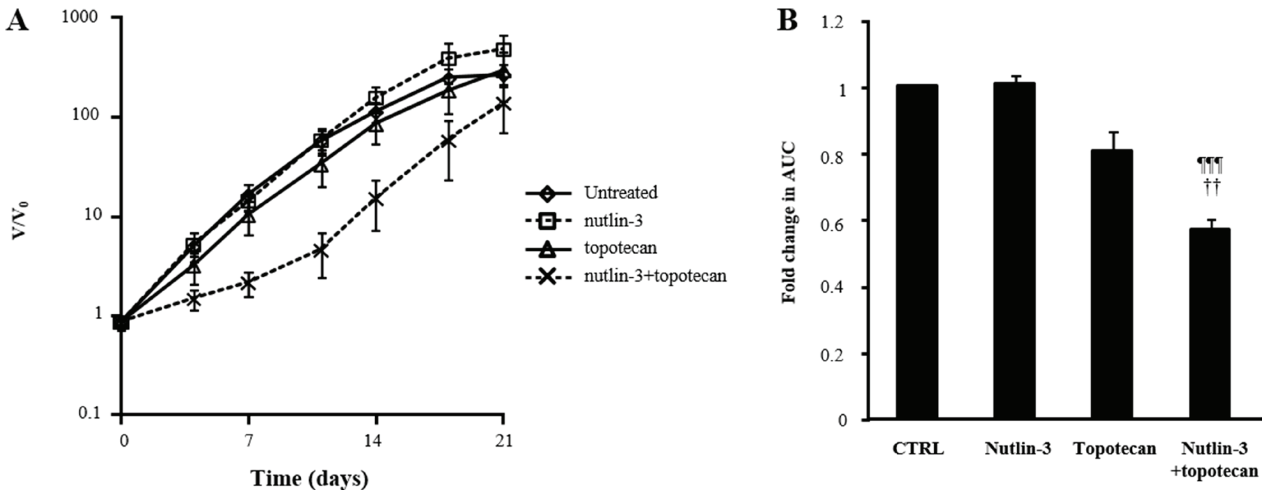

Supplementary Figure 3: The effect of reduced concentrations of nutlin-3 and topotecan on the growth of spheroids derived from CHLA-15 neuroblastoma cells. (**A**) CHLA-15 spheroids were treated with 5 µM nutlin-3 and 5 µM topotecan alone or in combination for 24 h. The volume, at time t, was calculated as described in Materials and Methods then divided by their initial volume at t0 to obtain the fold increase in volume (V/V_0). (**B**) The median AUC values resulting from each treatment were statistically compared with each other using Mann-Whitney pairwise comparisons. The symbol ¶ indicates a comparison with nutlin-3 alone and the symbol † indicates a comparison with topotecan alone. Two symbols indicate $P < 0.01$ and three symbols indicate $P < 0.001$.

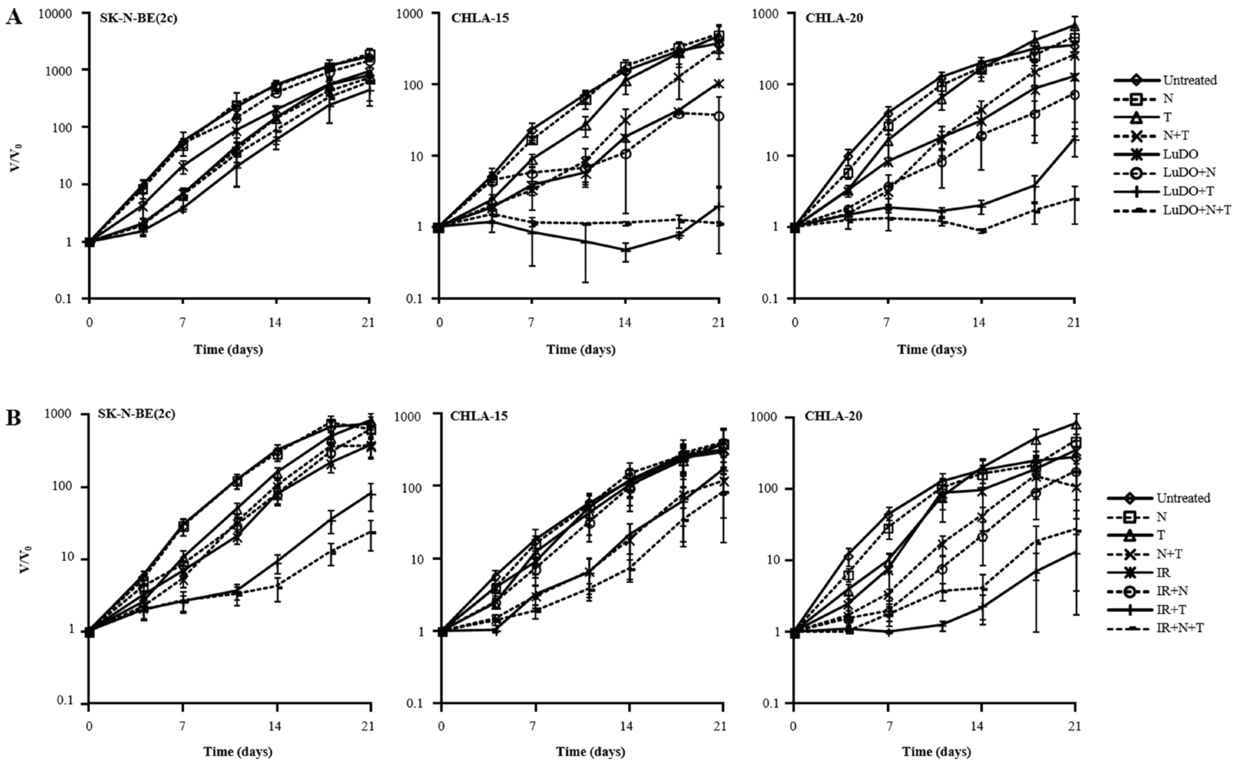

Supplementary Figure 4: The spheroid growth curves resulting from treatment with nutlin-3 and topotecan alone or in combination with ^{177}Lu-DOTATATE or X-irradiation. (**A**) SK-N-BE(2c) and CHLA-20 spheroids were treated with 10 µM nutlin-3 (N) and 10 µM topotecan (T) alone or in combination for 24 h. CHLA-15 spheroids were treated with 5 µM nutlin-3 and 5 µM topotecan alone or in combination for 24 h. ^{177}Lu-DOTATATE (LuDO) treatment consisted of exposure for 8 h to 5 MBq/ml. For combination treatments, the schedule of administrations of nutlin-3, topotecan and ^{177}Lu-DOTATATE is shown in Figure 5A. (**B**) SK-N-BE(2c) and CHLA-20 spheroids were treated with 10 µM nutlin-3 and 10 µM topotecan alone or in combination for 24 h. CHLA-15 spheroids were treated with 5 µM nutlin-3 and 5 µM topotecan alone or in combination for 24 h. SK-N-BE(2c), CHLA-15 and CHLA-20 spheroids were X-irradiated (IR) with 3 Gy, 1 Gy and 3 Gy, respectively. For combination treatments, the schedule of administrations of nutlin-3, topotecan and X-irradiation is shown in Figure 5B.
